# Identification of hub genes and immune cell infiltration characteristics in chronic rhinosinusitis with nasal polyps: Bioinformatics analysis and experimental validation

**DOI:** 10.3389/fmolb.2022.843580

**Published:** 2022-08-17

**Authors:** Yangwang Pan, Linjing Wu, Shuai He, Jun Wu, Tong Wang, Hongrui Zang

**Affiliations:** ^1^ Department of Otolaryngology Head and Neck Surgery, Civil Aviation General Hospital (Peking University Civil Aviation School of Clinical Medicine), Bejing, China; ^2^ Department of Otolaryngology Head and Neck Surgery, Beijing Tongren Hospital, Capital Medical University, Beijing, China

**Keywords:** nasal polyps, hub genes, immune cell infiltration, bioinformatics analysis, experimental validation

## Abstract

The aim of our study is to reveal the hub genes related to the pathogenesis of chronic rhinosinusitis with nasal polyps (CRSwNP) and their association with immune cell infiltration through bioinformatics analysis combined with experimental validation. In this study, through differential gene expression analysis, 1,516 upregulated and 1,307 downregulated DEG were obtained from dataset GSE136825 of the GEO database. We identified 14 co-expressed modules using weighted gene co-expression network analysis (WGCNA), among which the most significant positive and negative correlations were MEgreen and MEturquoise modules, containing 1,540 and 3,710 genes respectively. After the intersection of the two modules and DEG, two gene sets—DEG-MEgreen and DEG-MEturquoise—were obtained, containing 395 and 1,168 genes respectively. Through GO term analysis, it was found that immune response and signal transduction are the most important biological processes. We found, based on KEGG pathway enrichment analysis, that osteoclast differentiations, cytokine–cytokine receptor interactions, and neuroactive ligand–receptor interactions are the most important in the two gene sets. Through PPI network analysis, we listed the top-ten genes for the concentrated connectivity of the two gene sets. Next, a few genes were verified by qPCR experiments, and *FPR2, ITGAM, C3AR1, FCER1G, CYBB* in DEG-MEgreen and *GNG4, NMUR2,* and *GNG7* in DEG-MEturquoise were confirmed to be related to the pathogenesis of CRSwNP. NP immune cell infiltration analysis revealed a significant difference in the proportion of immune cells between the NP group and control group. Finally, correlation analysis between target hub genes and immune cells indicated that *FPR2* and *GNG7* had a positive or negative correlation with some specific immune cells. In summary, the discoveries of these new hub genes and their association with immune cell infiltration are of great significance for uncovering the specific pathogenesis of CRSwNP and searching for disease biomarkers and potential therapeutic targets.

## Introduction

Chronic rhinosinusitis (CRS) is a common disease in departments of otolaryngology, with a global incidence of 5%∼12% (Fokkens et al., 2020), causing a huge economic burden on many patients. According to EPOS 2020 guidelines, chronic rhinosinusitis with nasal polyps (CRSwNP) is considered a primary CRS associated with type 2 immune response (Fokkens et al., 2020). CRSwNP is a chronic inflammatory disease of sinus mucosa, which usually manifests as nasal obstruction, runny nose, and olfactory disturbance (Kwah and Peters, 2019). Because the pathogenesis of CRSwNP has not yet been fully clarified and is prone to recurrence, it is particularly urgent to better understand its molecular and genetic pathological mechanisms and promote the research and development of targeted drugs. Many studies have found that the role of genetic factors (Liu et al., 2020; Tubita et al., 2020) in CRSwNP patients cannot be ignored. Therefore, this study will explore the pathogenesis of CRSwNP from the perspective of genes.

With the development of the new generation of high-throughput sequencing technology, many gene databases have stored a large amount of genetic information about diseases (Barrett et al., 2013; Shimoyama et al., 2015; Sarkans et al., 2021). This lays a foundation for us to quantitatively research the biological process of disease by constructing a gene expression network system. Weighted gene co-expression network analysis (WGCNA) is a bioinformatics analysis method used to systematically study complex relationships between genes and disease phenotypes across different groups of samples. The significant advantage of WGCNA is that it can integrate decentralized gene expression data into co-expression modules to provide phenotypic features of interest (Zhang and Horvath, 2005; Zhao et al., 2010).

In this study, we constructed WGCNA based on the high-throughput sequencing dataset GSE136825 and explored the association between immune cell infiltration and hub genes. Our study identified hub genes and immune cell infiltration characteristics in CRSwNP through comprehensive bioinformatics analysis and experimental validation, providing strong evidence for identifying candidate biomarker genes and future drug therapeutic targets.

## Materials and methods

### Collection of high-throughput sequencing data

The National Center for Biotechnology Information (NCBI) Gene Expression Omnibus (GEO, http://www.ncbi.nlm.nih.gov/geo/) database stores many high quality gene expression datasets. We downloaded a large-sample high-throughput sequencing dataset GSE136825 on CRSwNP submitted by Andiappan AK, Guan W and based on the GPL20301 Illumina HiSeq 4000 (*Homo sapiens*) platform from the GEO database—it included 42 nasal polyps (NP) and 28 normal inferior turbinate (IT) samples. All CRSwNP patients had bilateral NP; those with antrochoanal polyps, fungal sinusitis and/or recurrent lower airway infections were excluded. None of the participants had CRS without NP or aspirin-exacerbated respiratory diseases (Peng et al., 2019).

### Screening of differentially expressed genes

Statistical software RStudio (Version 1.3.959, https://rstudio.com/) and Bioconductor packages (http://www.bioconductor.org/) were used for bioinformatics analysis of NP samples and normal IT samples. We firstly collated the dataset of raw data and made it accord with the RStudio software input file format, using limma, ggplot2, and pheatmap R package to map the heat maps and volcanic DEG. The adjusted *p* values <0.05 and | logFC | > 1 are considered statistically significant.

### Weighted gene co-expression network analysis

As a systematic bioinformatics analysis method, we used the Rstudio weighted gene co-expression network analysis (WGCNA) package (https://cran.r-project.org/web/packages/WGCNA/) in WGCNA. The gene expression matrix of the whole transcriptome was firstly examined to see if there were any missing values; if there were, these were filled. The NP and normal samples were then clustered. The soft threshold capability was determined by network topology analysis, and the co-expression similarity and adjacency of genes were calculated using the soft threshold capability. After that, the adjacency relationship was transformed into a topological overlap matrix (TOM) which was used for gene hierarchical clustering. The dynamic shear algorithm was applied to module recognition and the similar modules were clustered and merged. Finally, the gene-module tree diagram and the module-trait relationship diagram were outputted.

### Intersection of DEG and WGCNA

After WGCNA, the Venndiagram software package was used to intersect the results of DEG and WGCNA to obtain the NP-related genes in which we were interested. In the module-trait relationships diagram, the modules with the most significant positive and negative correlations were found and the positive and negative correlation module genes were intersected with DEG to obtain two Venn diagrams.

### GO term and KEGG pathway enrichment analysis

Firstly, the org.hs.eg. db R software package was used to convert the gene names of interested into gene IDs that could be recognized by the R language. Then, the R software packages Clusterprofiler, Enrich Plot and GGPLOT2 were used to conduct GO Term enrichment analysis on the genes of interest, including biological processes (BP), cell components (CC), and molecular functions (MF), in order to explore the biological significance of the genes of interest. KEGG enrichment analysis was then performed on the genes of interest to identify the key pathways closely related to the occurrence and development of CRSwNP. The adjusted *p* value < 0.05 is considered statistically significant.

### Protein–protein interaction network analysis

PPI network analysis helped identify the hub genes and key gene modules involved in the occurrence and development of CRSwNP from the interaction level. PPI information for the genes of interest was obtained from the Search Tool for Interacting Genes/Protein (STRING) database (http://www.string-db.org/). Next, we used Cytoscape software to build a PPI visual network. Finally, the Cytohubba plug-in was used in Cytoscape to select the top-ten genes with the highest connectivity from the genes of interest as the hub genes of the network to visualize them.

### Experimental validation of qRT-PCR analysis

The ten genes with the highest degree of connectivity (listed in Table 1) were selected for experimental validation. Our study included eight NP samples and eight normal samples. All participants signed an informed consent form prior to participating in the study which was approved by the Ethics Committee of Beijing Tongren Hospital, Capital Medical University. Eight NP were collected from three male and five female patients with a mean age of 48.10 ± 10.90. Eight normal tissues (non-CRS uncinate process tissues) were distributed in seven males and one female with an average age of 43.60 ± 6.59. All had either functional endoscopic nasal surgery or uncinate process resection. All CRSwNP subjects met the diagnostic criteria of United States and European guidelines. None had taken glucocorticoids, antibiotics, or antihistamines in the four weeks prior. Patients with fungal sinusitis, posterior nostril polyps, asthma, aspirin intolerance, allergic rhinitis, or smoking were excluded. The basic clinical data of all participants in this study are listed in Table 2. Tissue RNA stored in the RNAlater solution was extracted using a total RNA extraction kit (Soleabal, R1200) as recommended by the biological manufacturer; cDNA was synthesized using a universal reverse transcription kit (Yisheng Bio, 11141ES60). A real-time quantitative fluorescence PCR analyzer (Molarray, MA-6000) was used for qRT-PCR analysis using real-time PCR quantitative fluorescence kit (Yishengbiao, 11201ES08), and all PCR was repeated three times. GAPDH gene expression was used as a standardized endogenous control. We used a standard ΔΔCt method to calculate relative gene expressions by Roche LightCycler 480 software. A set of primers and probes were designed and improved for these genes (shown in Table 1).

**TABLE 1:** The genes and primers used for experimental validation by qRT-PCR.

Gene	Module	Primer (F)	Primer (R)
FPR2	MEgreen	ACA​CGC​ACA​GTC​ACC​ACC​ATC​T	AGC​AAG​AAT​CCA​AGG​TCC​GAC​GAT
ITGAM	MEgreen	ACC​TCG​CAT​AAC​CAC​CTC​CTG​AT	TGT​CCT​TGT​ATT​GCC​GCT​TGA​AGA​A
C3AR1	MEgreen	TTG​TTG​TCG​TGT​GGT​GTT​GAT​GGT	ACT​CAG​TCT​CAT​GGC​TTC​TTG​TCT​TC
FCER1G	MEgreen	CAG​GAA​CCA​GGA​GAC​TTA​CGA​GAC​T	AGA​GAA​GAA​GGG​TGG​GAC​AAG​AGA​G
CYBB	MEgreen	CTT​CGC​ATC​CAT​TCT​CAA​GTC​AGT​CT	CAG​CCA​GTG​AGG​TAG​ATG​TTG​TAG​C
ITGB2	MEgreen	ACA​ACA​ACT​CCA​TCA​TCT​GCT​CAG​G	GCC​ACG​ACC​ACT​ACA​CTC​AAC​AC
GNG4	MEturquoise	TGG​TAG​TCA​TAC​AGC​AAG​GCA​GGT	TAG​GAA​GAT​AGG​TGG​AGG​CGG​AGA
NMUR2	MEturquoise	TTC​CAC​TAT​CCT​AAC​TGC​CTC​ATG​C	TTA​TGC​CTG​TAG​ACT​GCT​GCC​AAG
GNG7	MEturquoise	TCC​TTC​TGC​GTG​GTC​CCT​TTG​A	TTA​ACT​TGG​AGA​TGG​ATG​CGT​GGC
AGT	MEturquoise	CTG​GAT​GTT​GCT​GCT​GAG​AAG​ATT​GA	ACC​GAG​AAG​TTG​TCC​TGG​ATG​TCA

**TABLE 2:** The basic clinical data of all participants.

	CRSwNP	Normal
Number of subjects	8	8
Sex, male/female	3/5	7/1
Age (y)	48.10 ± 10.90	43.60 ± 6.59
Duration (y)	2.8 (0.5–7.2)	NA
Endoscopic appearance (Lund‐Kennedy score)	12.88 ± 2.90	NA

### Nasal polyps immune cell infiltration analysis

To compare the differences in immune cell infiltration between NP and control tissues, we analyzed NP immune cell infiltration using the ggpubr R package (Zhang et al., 2020) and preprocessCore (Yan et al., 2016) to obtain the levels of immune cell infiltration in each sample. In addition, we assessed the infiltration levels of immune cells in both groups. A heat map, a violin diagram, and a correlation matrix were used to represent the difference results. *p* < 0.05 was considered statistically significant.

### Correlation analysis between target hub genes and immune cells

To reveal the association between immune cell infiltration and the expression level of target hub genes, Pearson correlation between gene expression and immune cell fraction was performed by R packages reshape2, ggpubr, and ggExtra (Zhang et al., 2020). At first, the gene expression matrix and the list of immune cell infiltration results were read, with the data being consolidated and normalized. Next, the correlation test of various immune cells was calculated in cycle and a correlation scatter diagram was drawn. Finally, we used a lollipop diagram to visualize the correlation between target hub genes and immune cells.

## Result

### Identification of DEG

**FIGURE 1:**
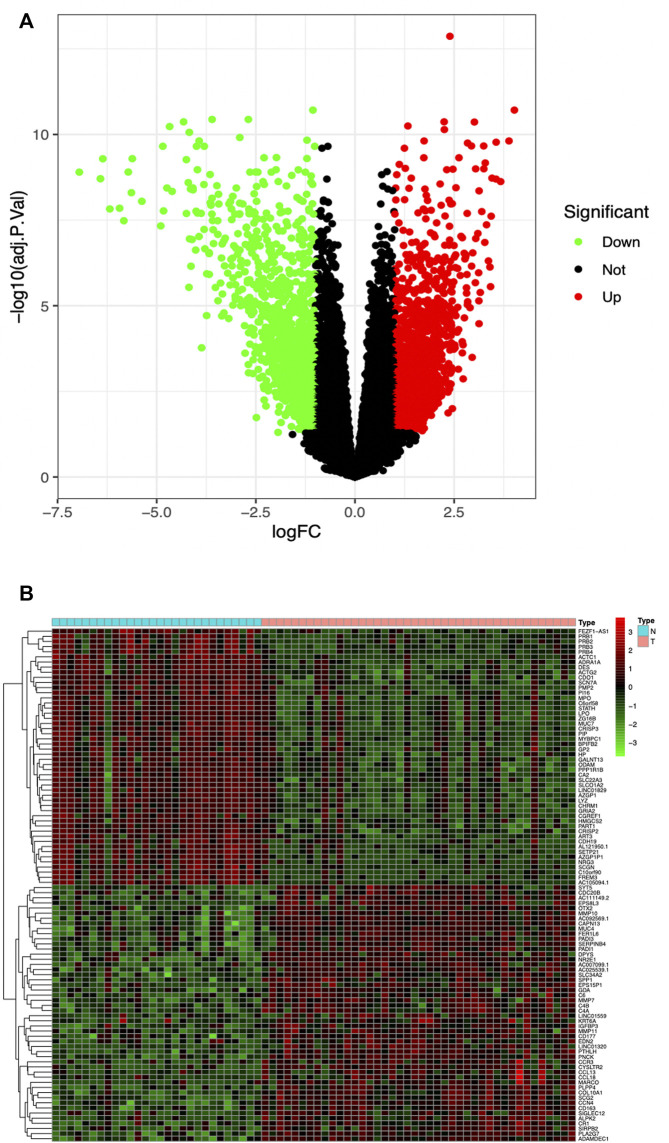
**(A)** Volcano plot of 2823 DEG in GSE136825, Red DEG with fold change >2; green DEG with fold change <2. **(B)** Heatmap of the top-50 DEG with fold change >2. Red upregulated DEG; green downregulated DEG. Small blue squares represent Normal group, the pink represent CRSwNP group.

The high-throughput sequencing dataset GSE136825 was downloaded from the GEO database, consisting of 42 CRSwNP-NP samples and 28 non-CRS-IT samples; the data passed the initial quality control of RNA-SEQ. Through pretreatment of the dataset and the analysis of DEG, we obtained 2823 DEG (the adjusted *p* values <0.05 and |logFC|>1), including 1516 DEG which were significantly upregulated and 1307 DEG which were significantly downregulated (as shown in Figure 1A). The top-50 DEG with the most significant upregulation and downregulation was, respectively, displayed by a gene heat map (as shown in Figure 1B).

### WGCNA of whole transcriptome gene expression matrix

**FIGURE 2:**
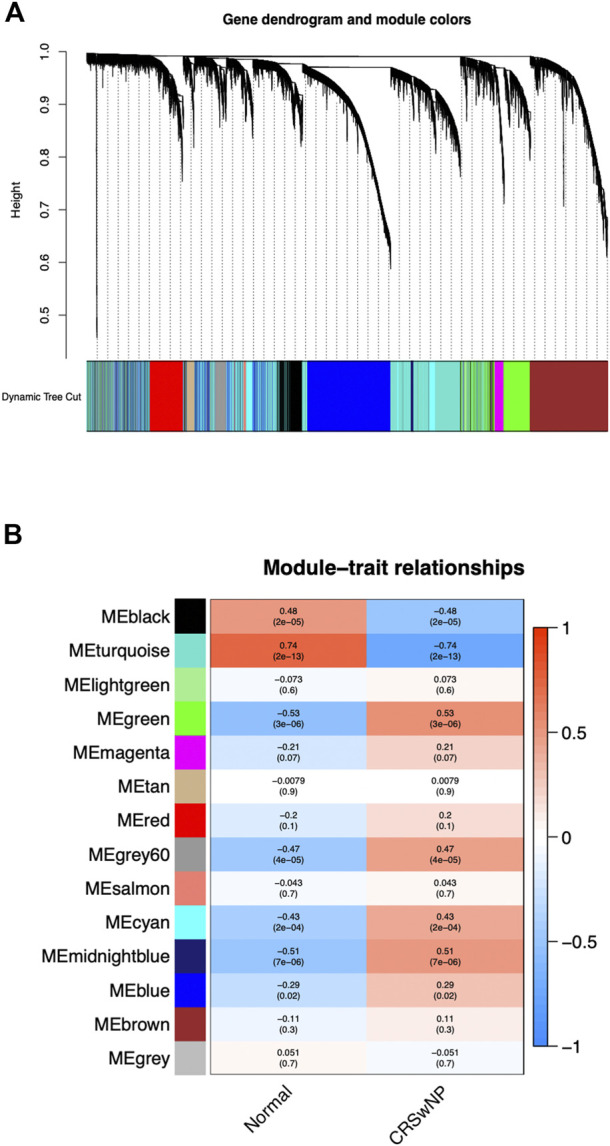
The constructed gene co-expression modules of CRSwNP by WGCNA in R. **(A)** Gene-module tree diagram. Each branch represents one gene, and every color below represents one co-expression module. **(B)** Module-trait relationship diagram. 14 modules were generated; the MEgreen (RS = 0.53, P = 3e-06) and MEturquoise (RS = −0.74, P = 2e-13) modules were most significantly related to CRSwNP.

Genes with the same expression tendency may also have certain correlations in some biological functions. Therefore, we constructed a weighted gene co-expression network on the dataset, analyzed the expression values of 21,734 corrected total transcriptome genes from 42 NP samples and 28 normal samples, mined out genes with similar expression profiles, and then clustered these genes and grouped them into the same module. We set the soft threshold power to 4 (scale-free *R* ^2^ = 0.90) to ensure a scale-free network. As shown in Figure 2A, a total of 14 gene modules were screened out. As could be seen from Figure 2B, the gene module with the most significant positive correlation with the CRSwNP group was MEgreen [Spearman correlation coefficient (RS) = 0.53, P = 3e-06], which contained 1,540 genes. The gene module with the most significant negative correlation was MEturquoise (RS = −0.74, P = 2e-13), which contained 3,710 genes.

### Intersection of DEG and the most significantly related module genes in WGCNA

**FIGURE 3:**
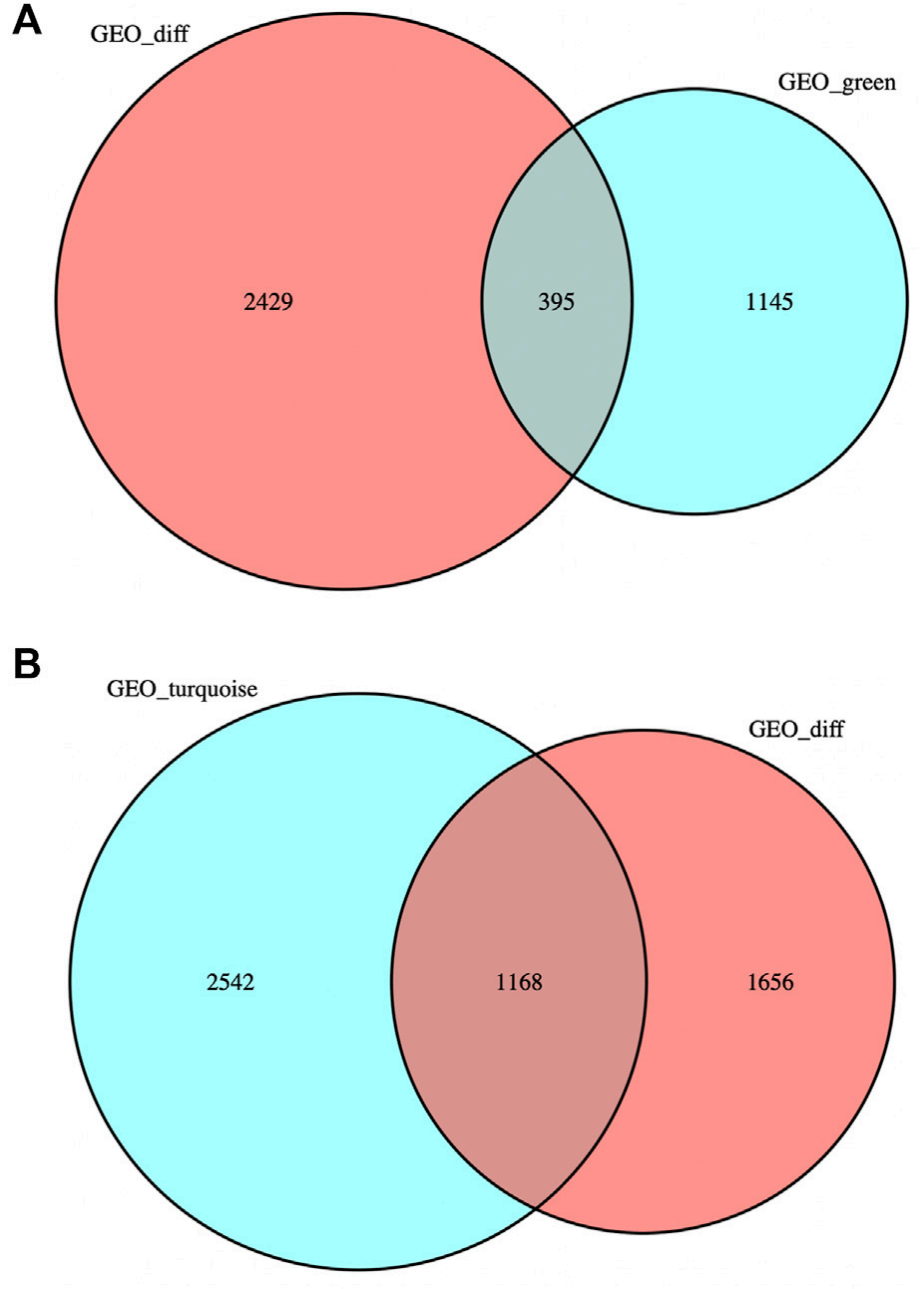
Venn diagrams. **(A)** DEG was intersected with the genes in MEgreen module: 395 genes were obtained. **(B)** DEG was intersected with the genes in MEturquoise module: 1,168 genes were obtained.

DEG was intersected with the genes in MEgreen and MEturquoise modules respectively, and the intersection gene sets obtained were named “DEG-MEgreen” and “DEG-MEturquoise”, containing 395 and 1,168 genes respectively. The gene sets were visualized to obtain two Venn diagrams (as shown in Figures 3A and B).

### CRSwNP function and pathway enrichment analysis of DEG-MEgreen and DEG-MEturquoise

**FIGURE 4:**
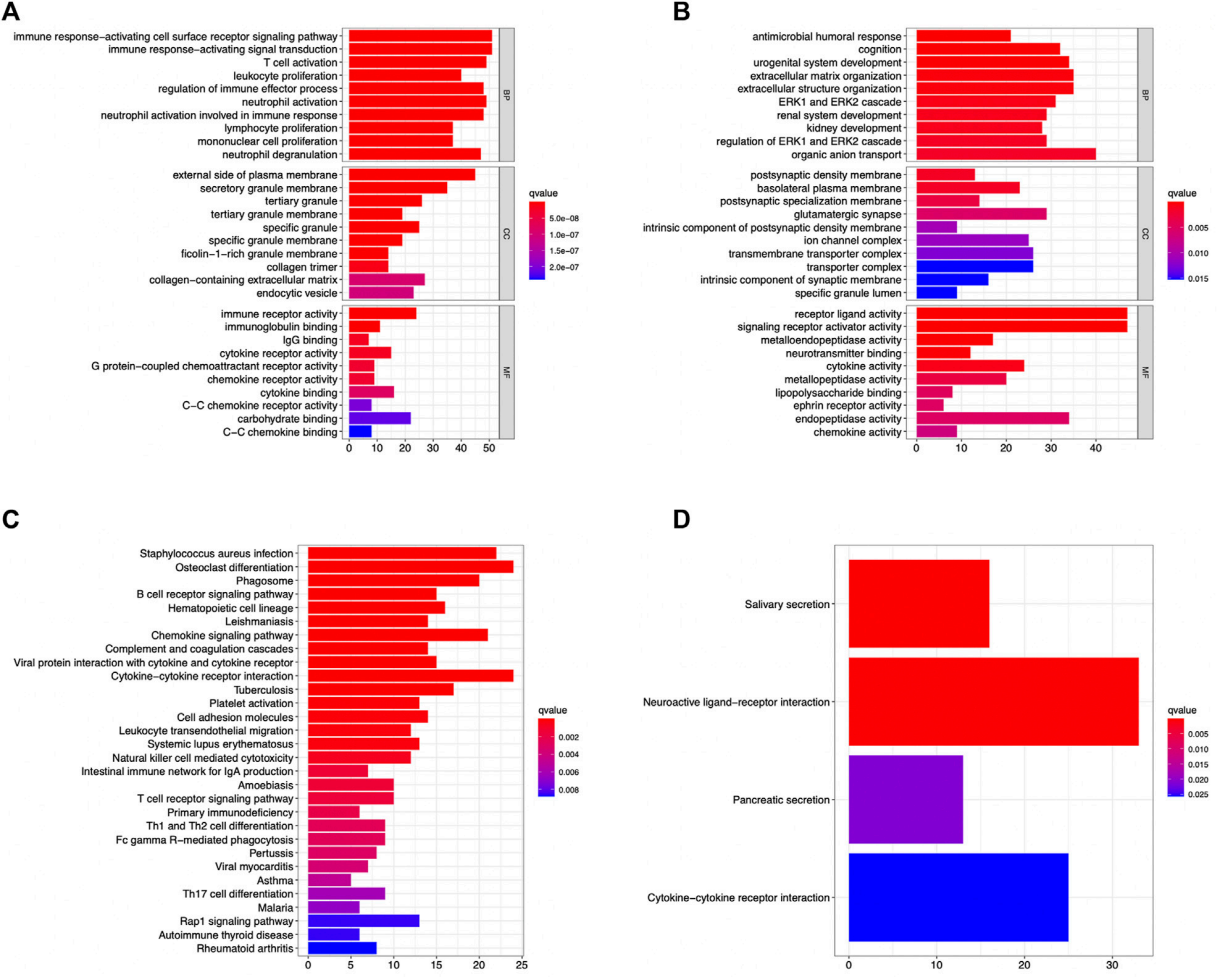
The GO enrichment and KEGG pathway enrichment analysis identified in the DEG-MEgreen **(A,C)** and DEG-MEturquoise **(B,D)** modules. The immune response and signal transduction were the main biological processes identified in DEG-MEgreen and DEG-MEturquoise, respectively. The majority of pathways identified in two modules were osteoclast differentiations, cytokine–cytokine receptor interactions, and neuroactive ligand–receptor interactions, respectively.

The GO enrichment and KEGG pathway enrichment DEG-MEgreen and DEG-MEturquoise were analyzed using RStudio. In DEG-MEgreen, GO enrichment analysis results showed that the BP functions include the immune response−activating cell surface receptor signaling pathway, immune response−activating signal transduction, T cell activation, and other immune biological processes. CC functions were related to the external side of plasma membrane, secretory granule membrane, and tertiary granule, and MF functions included immune receptor activity, carbohydrate binding, and cytokine binding (Figure 4A). In Deg-meturquoise, the results of GO enrichment analysis mainly identified the organic anion transport and extracellular matrix and structure organization involved in BP functions, the basolateral plasma membrane, the glutamatergic synapse and transmembrane transporter complex involved in CC functions, and the receptor ligand activity and the signaling receptor activator activity involved in MF functions (Figure 4B). In KEGG pathway analysis, the pathways identified in DEG-MEgreen were highly correlated with *Staphylococcus aureus* infection, osteoclast differentiation, chemokine signaling pathways, and cytokine–cytokine receptor interactions (Figure 4C). The pathways identified in DEG-MEturquoise were closely related to neuroactive ligand–receptor interaction, cytokine–cytokine receptor interactions, and salivary secretion (Figure 4D).

### Acquisition of hub genes by PPI network analysis

**FIGURE 5:**
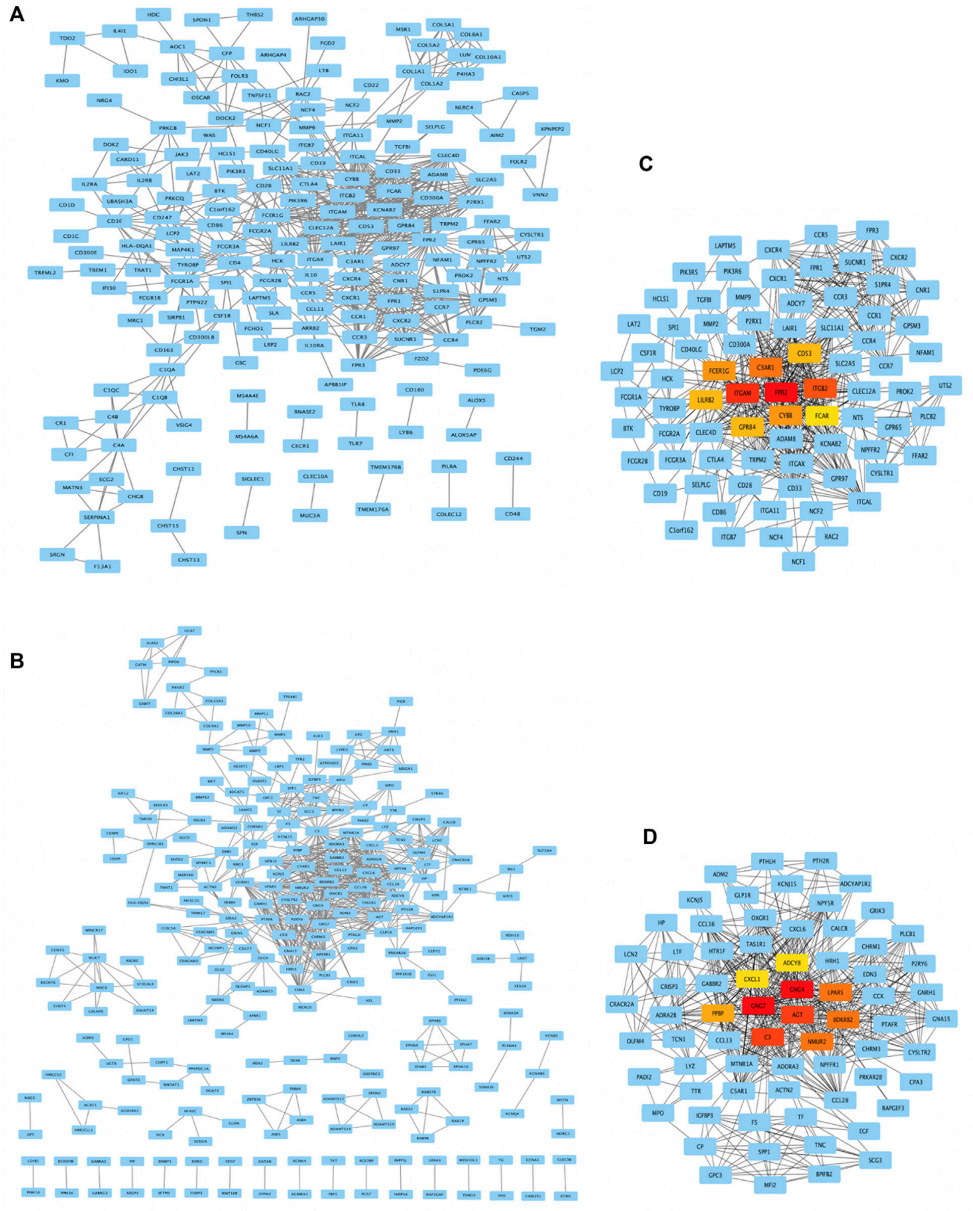
PPI network analysis and top-ten hub genes. **(A,B)** Genes from DEG-MEgreen and DEG-MEturquoise were put into PPI. **(C,D)** The top-ten connectivity genes in DEG-MEgreen and DEG-MEturquoise were visualized by Cytoscape. The ten highest degree genes in this PPI network were presented as red or orange nodes, where a node represents a gene and a line represents an interaction between nodes.

As shown in Figures 5A and B, genes from DEG-MEgreen and DEG-MEturquoise were put into PPI to analyze the connectivity of each protein interaction subnet, with genes with high connectivity identified as the hub genes of the network. As shown in Figures 5C and D, the top-ten connectivity genes in DEG-MEgreen and DEG-MEturquoise were visualized through Cytoscape software.

### Experimental validation

**FIGURE 6:**
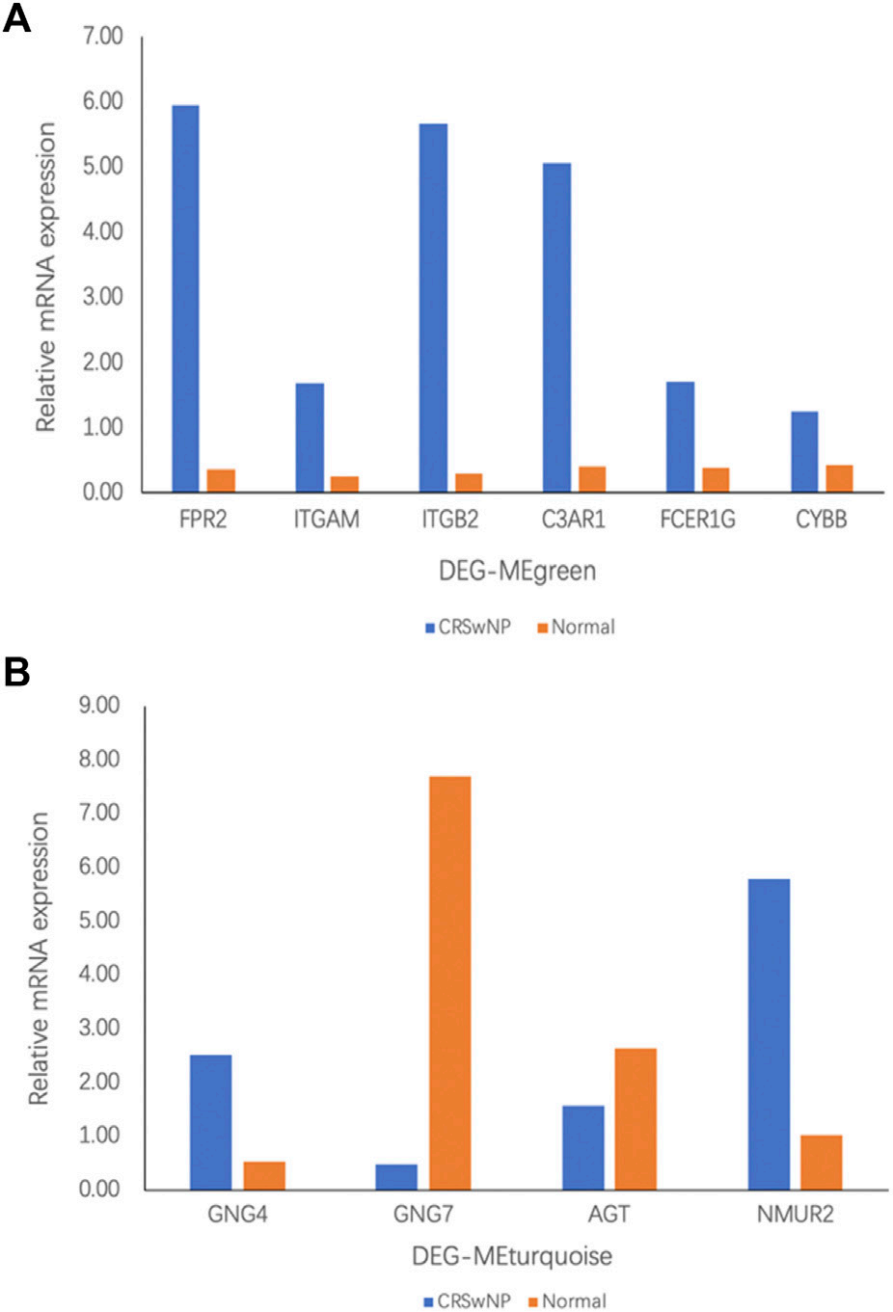
The mRNA expression in participants with CRSwNP (*n* = 8) and normal controls (*n* = 8) was assessed by qRT-PCR analysis. Fold change was calculated relative to normal controls. **(A)** mRNA expression in the DEG-MEgreen module. **(B)** mRNA expression in the DEG-MEturquoise module. All data are displayed as the mean, and *n* = 8 per group.

According to the above bioinformatics analysis results, our experiment verified the six genes with the highest connectivity in DEG-MEgreen and the four genes with the highest connectivity in DEG-MEturquoise. As shown in Figure 6A, Formyl peptide receptor 2 (*FPR2*) (16.78-fold), Integrin subunit alpha M (*ITGAM*) (6.83-fold), Complement C3a receptor 1 (*C3AR1*) (12.31-fold), Fc fragment of IgE receptor Ig (*FCER1G*) (4.31-fold), and Cytochrome b-245 beta chain (*CYBB*) (2.99-fold) were up-regulated in DEG-MEgreen. There were significant differences between CRSwNP and normal control group (*p* value < 0.05) while there was no statistically significant difference in Integrin subunit beta 2 (*ITGB2*) (18.81-fold, *p* value > 0.05). In DEG-MEturquoise, the expressions of G-protein subunit gamma 4 (*GNG4*) (4.75-fold) and Neuromedin U receptor 2 (*NMUR2*) (5.69-fold) were upregulated, and the expression of G-protein subunit gamma 7 (*GNG7*) (0.06-fold) was downregulated. Both of these showed significant differences among groups (*p* value < 0.05) while there was no statistically significant difference in Angiotensinogen (*AGT*) (0.59-fold, *p* value > 0.05), as shown in Figure 6B.

### The differential composition of infiltrating immune cells

**FIGURE 7:**
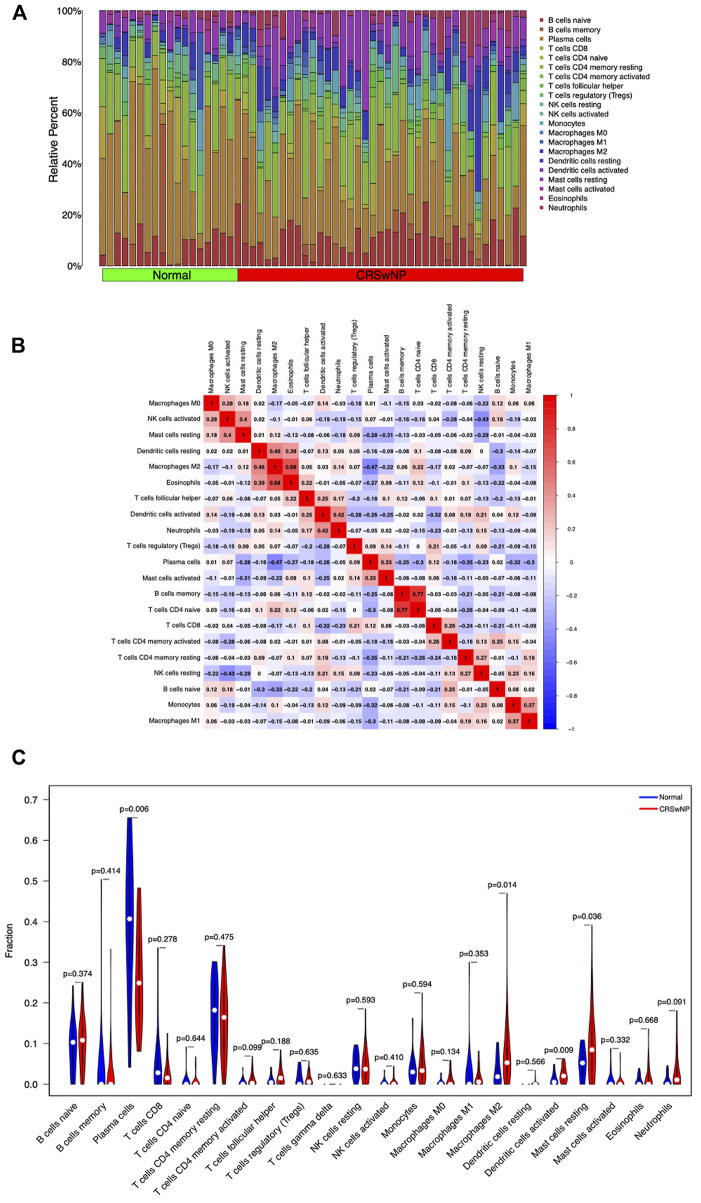
Differences and correlation of immune cell infiltration between the CRSwNP group and normal group. **(A)** Summary of immune infiltration in 22 immune cell subpopulations from 57 samples. **(B)** Correlation matrix of 22 immune cell infiltration in 57 samples. **(C)** Violin plot of differential expression of 22 infiltrating immune cells. Abscissa: immune cell types; ordinate: relative immune cell content; red: CRSwNP group; blue: Normal group.

Analysis of the infiltrating immune cell component by the CIBERSORT algorithm based on the GSE136825 dataset revealed immune cell classifications between the NP group and the normal group. The infiltration of each type of immune cell in each sample is summarized in Figure 7A and the co-expression correlation of various immune cells is displayed in Figure 7B. As illustrated in Figure 7C, in comparison with the control group, higher proportions of Macrophages M2, Dendritic cells activated, and Mast cells resting could be detected in the NP group, along with lower proportions of Plasma cells (*p*-value <0.05).

### Relationships between target hub genes and immune cells

**FIGURE 8:**
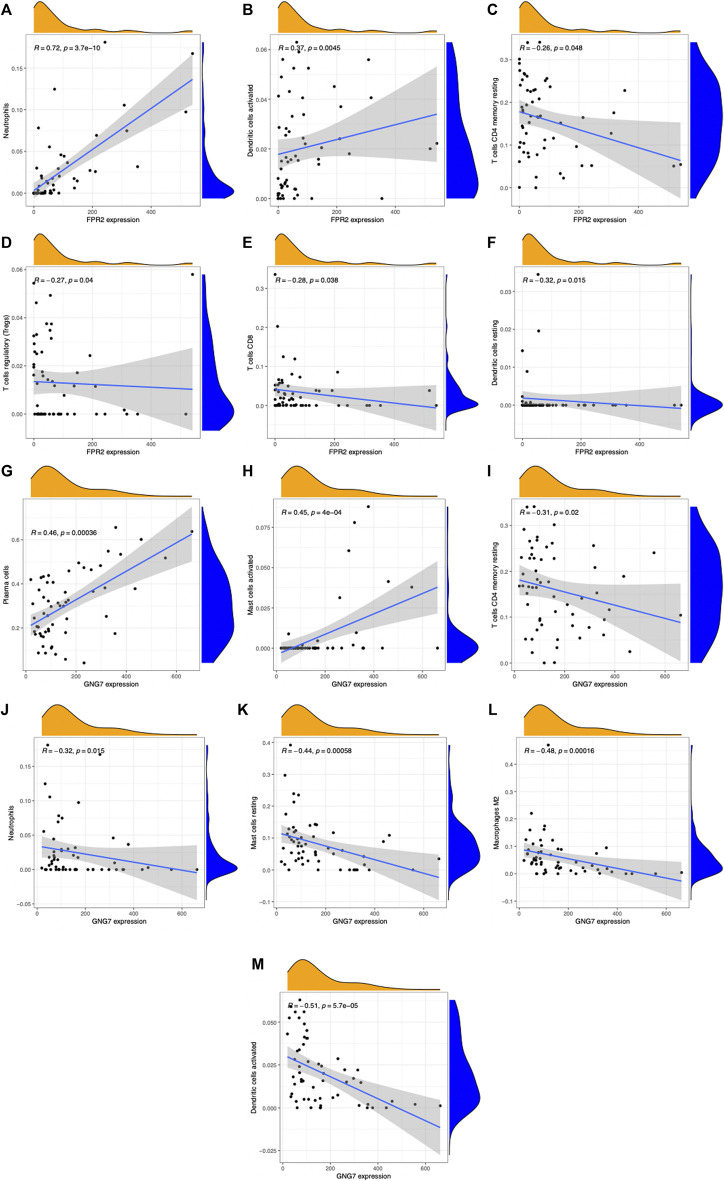
Scatter plots of correlation between *FPR2* or *GNG7* expression and different immune cell contents **(A–M)**. **(A)** Neutrophils, **(B)** dendritic cells activated, **(C)** T cells CD4 memory resting, **(D)** T cells regulatory (Tregs), **(E)** T cells CD8, and **(F)** dendritic cells resting. **(G)** Plasma cells, **(H)** mast cells activated, **(I)** T cells CD4 memory resting, **(J)** neutrophils, **(K)** mast cells resting, **(L)** macrophages M2, and **(M)** dendritic cells activated. R: correlation coefficient, *p* < 0.05 means a significant correlation.

**FIGURE 9:**
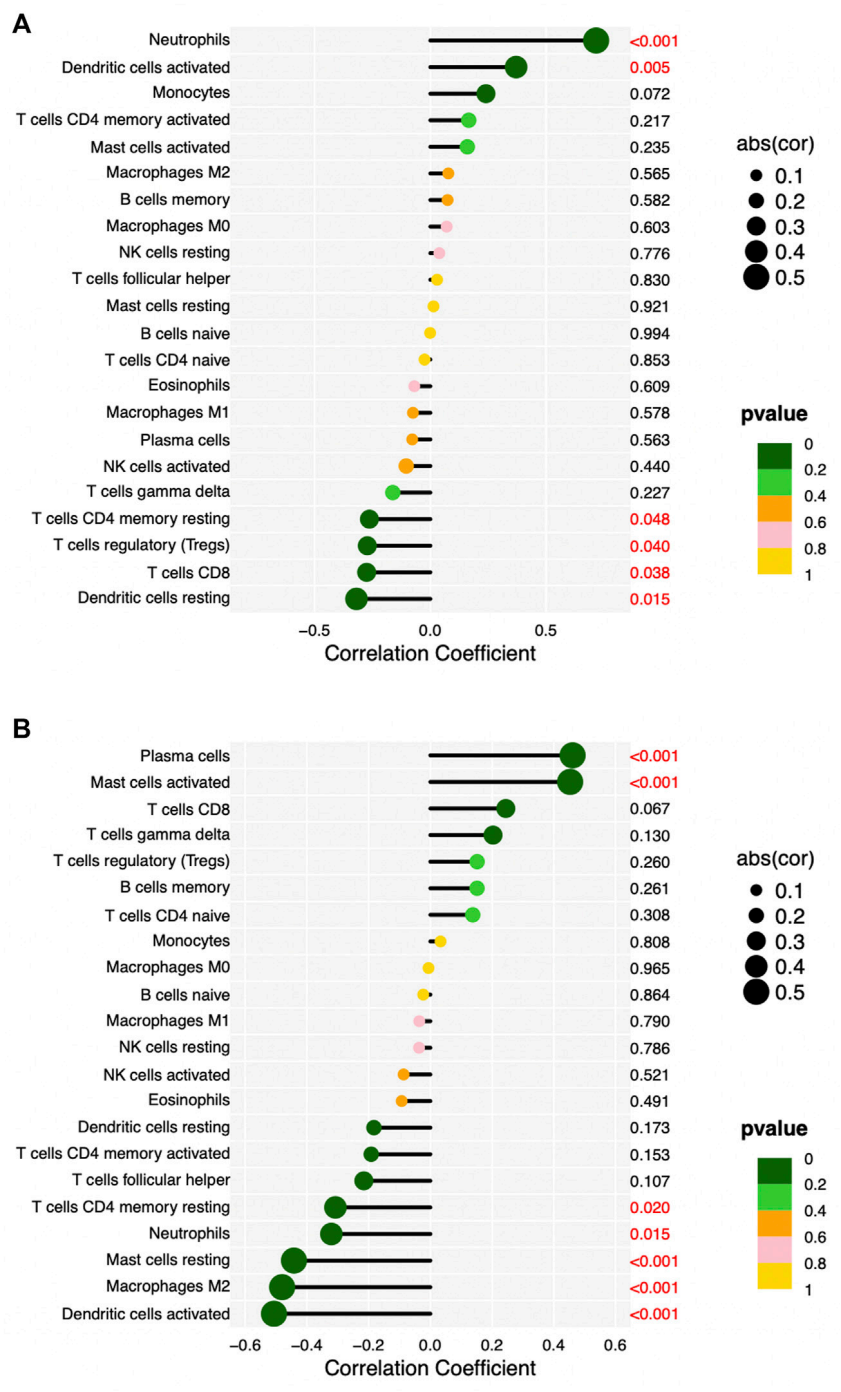
Lollipop diagram of correlation between *FPR2* **(A)** or *GNG7* **(B)** expression levels and 22 immune cell infiltrations. Circle size: absolute value of correlation coefficient; circle color: *p*-value of correlation test. *p* < 0.05 (red) indicates a significant correlation between *FPR2* **(A)** or *GNG7* **(B)** expression and immune cell contents.

We validated the RNA-seq results by qPCR for selected hub genes, among which *FPR2*(16.78-fold) and *GNG7* (0.06-fold) with the greatest fold difference presented a variety of correlations to immune cell infiltration. Figures 8 and 9 show the significant correlation between *FPR2* or *GNG7* and immune-infiltrating cells. *FPR2* had a positive correlation with Neutrophils and Dendritic cells activated (Correlation Coefficient > 0 and *p*-value < 0.05) and a negative correlation with T cells CD4 memory resting, T cells regulatory (Tregs), T cells CD8 and Dendritic cells resting (Correlation Coefficient < 0 and *p*-value < 0.05). Similarly, *GNG7* correlated positively with Plasma cells and Mast cells activated but negatively with T cells CD4 memory resting, Neutrophils, Mast cells resting, Macrophages M2, and Dendritic cells activated.

## Discussion

The clinical characteristics of CRSwNP include chronic inflammation of sinus mucosa, nasal obstruction, and growth of nasal polyps. Due to its refractory and recurrent nature, CRSwNP brings a heavy psychological pressure and economic burden to patients. There are, at present, few studies on the pathogenesis and phenotypic characteristics of CRSwNP at the gene level. In this study, we used WGCNA to identify the key modules and hub genes involved in the molecular mechanism of CRSwNP and identified two gene sets most related to CRSwNP: DEG-MEgreen and DEG-MEturquoise. Through GO term analysis, it was found that immune response and signal transduction are the most important biological processes in DEG-MEgreen and DEG-MEturquoise respectively. KEGG pathway enrichment analysis showed that osteoclast differentiation, cytokine–cytokine receptor interactions, and neuroactive ligand–receptor interaction are most important in DEG-MEgreen and DEG-MEturquoise, respectively. Through PPI network analysis, we listed the top-ten genes in the connectivity of DEG-MEgreen and DEG-MEturquoise. Next, a few genes were verified by qPCR experiments with the samples from our hospital; *FPR2, ITGAM, C3AR1, FCER1G, CYBB* in DEG-MEgreen, and *GNG4, NMUR2* and *GNG7* in DEG-MEturquoise were confirmed to be related to the pathogenesis and phenotype of CRSwNP. We then used immune cell infiltration analysis to find that higher proportions of Macrophages M2, Dendritic cells activated, and Mast cells resting could be detected in the NP group, along with lower proportions of Plasma cells. Finally, correlation analysis between target hub genes (*FPR2* and *GNG7*) and immune cells revealed significant relationships.

In this study, a total of 14 modules were mined by WGCNA, among which the MEgreen and Meturquoise modules correlated most with CRSwNP. Through traditional experimental methods and bioinformatics analysis, many studies in the past have shown that immune response and signal transduction play an important role in the pathogenesis of CRSwNP. Bassiouni et al. (2020) analyzed the whole transcriptome of nasal polyps by high-throughput sequencing and found that the B cell-mediated immune response of CRSwNP polyps is upregulated and the expression of genes related to epithelial morphogenesis and hemostasis is downregulated. Studies have shown that CRSwNP is driven by cytokines *IL-5* and *IL-13* produced by Th2 cells, type 2 innate lymphoid cells, and mast cells, while type 2 cytokines activate inflammatory cells associated with pathogenic mechanisms, including mast cells, basophils, and eosinophils (Schleimer, 2017). Recently, using a mouse NP model and human tissues, the Bae JS team (Bae et al., 2020) found that the Wnt signaling pathway may be involved in the pathogenesis of NP through epithelial cell to mesenchymal transformation (EMT). Therefore, the inhibition of Wnt signaling may be a potential treatment strategy for patients with CRSwNP. However, in our study, the GO term analysis of DEG-Megreen and DEG-Meturquoise proved that immune response and signal transduction are the most important biological processes of the two gene modules. These results indicated that WGCNA can predict the exact biological processes involved in the pathogenesis of CRSwNP, which provides guidance for further exploration of its genetic and molecular mechanisms.

It is worth noting that, in this study, the KEGG pathway analysis of both DEG-Megreen and DEG-Meturquoise indicated that the cytokine–cytokine receptor interaction plays a vital role in the occurrence and development of CRSwNP. In addition, the *S. aureus* infection, osteoclast differentiation, and the neuroactive ligand–receptor interaction are the unique biological roles of the two module genes, respectively. Lan et al. (2018) demonstrated for the first time that *S. aureus* can directly induce the release of epithelial-derived cytokines by binding Toll-like receptor 2 through the human nasal mucosal tissue model, thereby spreading the expression of type 2 cytokines in NP tissues. Liao et al. (2015) examined some cytokines and their receptors in NP tissue through rigorous experiments and found that the positive feedback loop between *TSLP, IL-33* and its receptor, and Th2 cytokines in eosinophilic CRSwNP may promote Th2 bias inflammation. Two studies in NP mouse models have shown that osteoclast formation and differentiation are involved in the pathological tissue remodeling process of CRSwNP (Pan et al., 2018; Khalmuratova et al., 2020). Only one study has shown that CRSwNP can impair the olfactorial signaling pathway and neuroactive ligand receptor pathway by affecting the expression of “olfactorial receptor activity” and “channel activity” genes (Xiong et al., 2020). These clinical and animal experimental results provided the evidential support for WGCNA to be used to explore the specific pathogenic mechanism of CRSwNP.

In the two gene sets of DEG-Megreen and DEG-Meturquoise, we listed ten hub genes that affect the pathogenesis of CRSwNP; our experiments proved that, in DEG-Megreen, *FPR2, ITGAM, C3AR1, FCER1G,* and *CYBB* are most significantly positively correlated with CRSwNP. In DEG-Meturquoise, *GNG4, NMUR2,* and *GNG7* were most significantly negatively correlated with CRSwNP. To date, no studies have reported the involvement of these genes in the occurrence and development of CRSwNP. A recent study has confirmed that the formyl peptide receptor agonist *Ac2-26* exerts an anti-inflammatory effect in a mouse model of bacterial meningitis through the formamide peptide receptor 2 (*FPR2*) (Rüger et al., 2020). *FPR2* agonists could stimulate the resolution of inflammation by inhibiting neutrophil chemotaxis and stimulating macrophage phagocytosis, which may be the key to the treatment of chronic inflammatory diseases (Asahina et al., 2020). Odobasic et al. (2018) investigated the effects of *FPR2* activation on output T cell response and fibroblast-like synovial cell expansion in a rheumatoid arthritis model and found that the *FPR2* activator reduced the proliferation and survival of CD4 T cells in lymph nodes and increased the production of the protective cytokines IFNγ and anti-inflammatory IL-4. Therefore, the high expression of *FPR2* may activate the immune regulatory system of CRSwNP patients through similar biological behavior. It has been reported that *C3AR1* may be a major regulator of microglia reactivity and neuroinflammatory functions (Harder et al., 2020) and that *C3AR1* activates by the dual ligand *TLQP-21* and *C3a* being involved in microglia signaling under pathological conditions—a new neuroimmune signaling pathway (Doolen et al., 2017). Chauhan P’s team (Chauhan et al., 2017) observed a significant delay in microglia polarization to the IFNγ and IL-4-activated M2 phenotype in the absence of the Fcγ receptor for IgG in murine cytomegalovirus-infected *FCER1G* and *FCGR2B* knockout mice. The results of this study provide gene therapy ideas for inhibiting neuroinflammation caused by virus infection. *NMUR2* is a neuropeptide receptor whose ligand *NMU* is involved in a variety of physiological processes, such as the regulation of food intake and other behaviors (De Prins et al., 2018). Therefore, it is expected to be a targeted drug for the treatment of diabetes and obesity (Kanematsu-Yamaki et al., 2017). Upregulation of G-protein subunit γ4 (*GNG4*) is associated with many cancers, such as colon cancer (Song et al., 2020), triple-negative breast cancer (Zhang et al., 2020), thymic carcinoma (Kishibuchi et al., 2020), and hepatocellular carcinoma (Ding et al., 2019). It was found that G-protein γ7 (*GNG7*) is a tumor suppressor gene in the progression of renal cell carcinoma (RCC) and is a novel replacement gene in the treatment of RCC (Xu et al., 2019). Demokan et al. (2013) found that *GNG7* is a highly specific promoter methylation gene associated with head and neck squamous cell carcinoma (HNSCC) and that downregulation of *GNG7* expression is a common event in HNSCC (Hartmann et al., 2012). These results indicate that WGCNA could be used to reveal new pathogenic genes and provide reliable theoretical evidence for experimental studies in the future.

Our study showed that a great quantity of disease-related genes and modules could be mined through WGCNA. The accuracy and reliability of WGCNA are confirmed through experimental validation and immune cell infiltration and correlation analyses. In the field of CRSwNP research, there are few innovative studies that apply WGCNA to high-throughput sequencing data, and our experimental results have been satisfactory as verified. However, there are no more large-sample datasets, and a lack of prognosis, follow-up, and other clinical information, which are the limitations of our study.

In conclusion, we identified some hub genes and key modules that were closely related to the molecular mechanism of CRSwNP, conducted immune cell infiltration analysis and correlation analysis for the target hub genes through integrated bioinformatics analysis, together with our experimental validation. For example, *FPR2, ITGAM, C3AR1, FCER1G, CYBB, GNG4, NMUR2,* and *GNG7* had been proved to be highly correlated with the pathogenesis of CRSwNP. Further study of these new genes is of great significance for uncovering the pathogenesis of CRSwNP and the search for disease biomarkers and potential therapeutic targets.

## Data Availability

Publicly available dataset was analyzed in this study. This data can be found here: https://www.ncbi.nlm.nih.gov/geo/query/acc.cgi?acc=GSE136825 and the original contributions presented in the study are included in the article/supplementary material, further inquiries can be directed to the corresponding authors.

## Abbreviations

BP, biological processes; CC, cell components; CRS, chronic rhinosinusitis; CRSwNP, chronic rhinosinusitis with nasal polyps; GEO, Gene Expression Omnibus; HNSCC, head and neck squamous cell carcinoma; IT, inferior turbinate; MF, molecular functions; NCBI, National Center for Biotechnology Information; NP, nasal polyps; PPI, protein–protein interactions; RCC, renal cell carcinoma; TOM, topological overlap matrix; WGCNA, weighted gene co-expression network analysis.
